# Comparing Management Strategies for Thoracolumbar Injury Classification and Severity Score of 4 (TLICS = 4) in the Pediatric Population: A Single-Institutional Experience

**DOI:** 10.3390/children11121529

**Published:** 2024-12-17

**Authors:** Jose Castillo, Michael Nhien Le, Khadija Soufi, James Zhou, Edwin Kulubya, Anzhela Moskalik, Yashar Javidan, Julius O. Ebinu

**Affiliations:** 1 Department of Neurological Surgery, University of California, Davis, Sacramento, CA 95817, USA; miqle@ucdavis.edu (M.N.L.); khsoufi@ucdavis.edu (K.S.); james.x1.zhou@kp.org (J.Z.); esk2120@cumc.columbia.edu (E.K.); admoskalik@ucdavis.edu (A.M.); 2 Department of Orthopedic Surgery, University of California, Davis, Sacramento, CA 95817, USA; yjavidan@ucdavis.edu; 3 Department of Surgery, Division of Neurosurgery, Queen’s University, Kingston, ON K7L 3N6, Canada; julius.ebinu@queensu.ca

**Keywords:** pediatric fractures, thoracolumbar, spine trauma, minimally invasive surgery

## Abstract

Background: Thoracolumbar (TL) fractures are uncommon injuries in the pediatric population. Surgery is recommended for TL fractures with significant deformity, posterior ligamentous complex disruption, or neurological compromise. The Thoracolumbar Injury Classification and Severity Scale (TLICS) has been validated in pediatric populations and serves as a valuable tool for guiding treatment decisions. However, there remains a lack of clarity regarding the appropriate treatment for patients with a TLICS of 4. While conservative and surgical techniques have been described, most studies focused on adult populations, and there is no consensus on the appropriate management in the pediatric population. We reviewed our institutional experience of TL fractures in young children with TLICS of 4, managed both non-operatively and operatively. Methods: A retrospective review of a single institution’s experience managing pediatric patients (<18 years old) with TL fractures receiving a TLICS of 4 from 2015 to 2023 was conducted to determine the clinical outcomes following non-operative and operative treatment. Results: Among 11 pediatric patients, 4 were managed with bracing alone, primarily for posterior column fractures, using a thoracolumbar sacral orthosis (TLSO). Four patients underwent minimally invasive screw fixation (MISF), for Chance or posterior column fractures, with an average operative time of 143 min, blood loss of 29 cc, length of stay (LOS) of 9.8 days, and a follow-up interval of 6 months. Three patients received open posterior screw fixation (OPSF), most commonly for Chance fractures, with averages of 129 min operative time, 225 cc blood loss, 9.7 days LOS, and 4 months follow-up. Both MISF and OPSF utilized intra-operative imaging, with lower radiation exposure in the MISF group. One MISF patients had hardware failure evident by screw lucency on follow-up imaging. Conclusions: Bracing and surgery are safe management options for pediatric TL fractures receiving a TLICS of 4. MISF is an effective alternative treatment strategy, comparable to OPSF, with the advantage of reduced blood loss and radiation exposure. Further studies with age-matched cohorts and long-term outcomes may help determine the optimal management course.

## 1. Introduction

In children, spine trauma is frequently associated with motor vehicle collision (MVC), with 80% of injuries occurring in the cervical spine, followed by the thoracic (9%) and lumbar (5%) segments [1,2,3,4,5]. Compression fracture is the most common type of thoracolumbar (TL) fracture in children, with burst fractures more commonly associated with high-energy trauma and neurological compromise [4]. Traumatic spine injuries are a concerning cause of morbidity and mortality in pediatric patients, often presenting unique challenges due to the anatomical differences in children compared to adults. One notable example is the well-established fact that the posterior joint surfaces in young children are more horizontal than in adults. This anatomical characteristic increases the risk of anteroposterior displacements during injuries [6]. Failure to treat these spinal injuries appropriately can result in permanent spinal deformities and sagittal imbalance [7]. These injuries can also increase the chances of a permanent irreversible neurological injury in children.

Historically, TL fractures were traditionally managed non-operatively with the use of casting in cases of clean, bony fractures without significant soft tissue damage, or in neurologically intact patients [2,4,8,9,10,11]. However, non-operative management has been associated with high failure rates of up to 42% [12]. This high failure rate often requires subsequent interventions, which demonstrates the limitations of conservative approach in certain cases. As a result, surgical stabilization has become the recommended option, especially for patients presenting with kyphotic deformity, radiographic findings of instability, or severe injuries resulting in neurological compromise caused by compressive forces [13,14,15].

All these points speak to the importance of recognizing and managing these traumatic spine injuries early and appropriately. Classification systems providing detailed treatment recommendations have been well-established in adults but remain insufficient in the pediatric population [16]. In adults, one widely accepted thoracolumbar injury classification system is the Thoracolumbar Injury Classification and Severity Scale (TLICS) which is based on the injury mechanism, the integrity of the posterior ligamentous complex (PLC), and neurological status. While the TLICS has been validated in the pediatric population, limited literature is available on the appropriate management for those in the equivocal category of a TLICS of 4. The safety and efficacy of surgical treatment for unstable adult TL fractures are well-documented [8,10,13], but literature focusing on the optimal management in pediatric patients in this equivocal category remains limited [10,12,17]. This highlights the need for further investigation to determine the effective treatment tailored to pediatric population and whether a non-operative or operative approach is more ideal than the other.

We report a case series of pediatric patients with traumatic TL fractures treated at our level 1 trauma institution all classified within the TLICS of 4 category. We examined the clinical presentations, management strategies, and patient outcomes across 3 different treatment modalities, including conservative bracing, minimally invasive screw fixation (MISF), and open screw fixation (OPSF). Our study aims to provide insights into the advantages and limitations of each approach, contributing to the body of evidence guiding optimal care for pediatric patients with traumatic TL fractures that comprise a TLICS of 4.

## 2. Materials and Methods

An IRB-approved retrospective chart and radiographic review was conducted utilizing a database of pediatric patients (<18 years of age) who underwent treatment for traumatic TL fractures. This database included all pediatric traumatic TL fractures that were encountered at our institution by post-residency, fellowship-trained spine surgeons (4 orthopedic and 3 neurosurgery spine surgeons). Cases reviewed spanned from 2015 to 2023. This case series has been compiled and reported in accordance with the PROCESS Guidelines [18].

### 2.1. Variables

Demographic variables of interest were collected for all patients, including age, gender, height, weight, race, and ethnicity. Injury characteristics were analyzed to understand the various etiologies to determine which bracing, MISF, or OPSF would be most effective, including fracture type, procedure level, and number of levels of fusion performed.

Intra-operative variables such as operating room (OR) time, blood loss, screw length/diameter, radiation exposure via X-ray or O-ARM CT scan were collected to evaluate operative safety. Post-operative complications, spine alignment, and length of stay (LOS) were also reviewed to assess efficacy and follow-up safety of the 3 different treatment modalities with scheduled follow-up times in months.

### 2.2. Preoperative Surgical Decision Making

All pediatric patients were clinically assessed as per standard pediatric trauma guidelines, which included a full preoperative neurological examination and spine computed tomography (CT) imaging. The fracture morphologies assessed included unstable TL fractures such as Chance fractures or horizontal fractures comprising posterior column fractures which frequently lead to a TLICS of 4 on assessment.

The management strategy after assessment using TLICS and obtaining a value of 4 and deciding whether to manage operatively or non-operatively was guided by the expertise and experience of the on-call surgeon in managing TL fractures. The decision to manage minimally invasively with either a fluoroscopic-guided navigation technique or a robot-assisted (RA) percutaneous screw fixation technique was also based on the on-call surgeon’s familiarity with their use and the availability of the robot on the day of surgery. Only 2 patients received RA percutaneous instrumentation.

### 2.3. Treatment Technique

In this study, patients in the non-operative group were managed in a brace which included a thoracolumbar sacral orthosis (TLSO), or Risser cast that was worn when out of bed for 6–8 weeks.

In the MISF group, surgery involved fluoroscopic-guided, navigation (Medtronic, STEALTH navigation) or RA (Globus Excelsius GPS robot) percutaneous screw fixation. All surgeons performing percutaneous pedicle screw fixation used a lateral-to-medial trajectory. RA screw fixation was performed based on intraoperative screw trajectory planning from 3-dimensional images generated from intraoperative O-ARM fluoroscopy (Globus Excelsius GPS robot). All 3 imaging planes were used to plan the screw trajectory.

In the OPSF group, surgery involved only fluoroscopic-guided screw fixation. All surgeons performing pedicle screw fixation used a lateral-to-medial trajectory. In both the MISF and OPSF group, braces were used in the post-operative period for 6–8 weeks when out of bed.

### 2.4. Alignment

To evaluate spinal alignment, the Cobb angle at the fracture level was measured on radiographs at initial presentation, immediately post-treatment or with patients wearing the brace to determine the in-brace correction of kyphosis. Follow-up measurements were obtained to assess the maintenance of correction or progression of deformity over time. These measurements served as a quantifiable metric to evaluate the anatomical outcomes of the chosen treatment modalities. A change was only considered significant if the Cobb angle change was greater than 5 degrees when comparing to pre-operative measurements. Stable alignment was defined as having no exaggerated focal kyphosis around the fracture level. These thresholds represent clinically significant changes in spinal alignment that allow a standardized assessment of treatment efficacy.

### 2.5. Statistical Analysis

Descriptive statistics, including mean and total range, were collected and compiled using GraphPad Prism version 10 (La Jolla, CA, USA). Continuous outcomes were assessed using Mann-Whitney test for comparisons between two groups and the Kruskal Wallis test for comparisons among three groups with non-parametric data. Nominal data were analyzed using Fisher’s exact test as all cell counts were less than 5.

## 3. Results

### 3.1. Demographics

A total of 11 pediatric patients with TL fractures meeting the TLICS of 4 category was identified in our series. Seven patients were males, and four patients were females. The age range varied between 4 and 17 years-old and the average age was 11.9. 4 were managed with bracing, 4 with MSIF, and 3 with OPSF. Overall, 6 injuries were following an MVC, 1 after an automobile versus pedestrian, 2 after falls, and 1 after an all-terrain vehicle (ATV) or penetrating injury (Table 1). Several patients had associated injuries including pneumothoraces or rib fractures (*n* = 3), abdominal injuries (*n* = 4), and other facial (*n* = 2), and extremity fractures (*n* = 4).

### 3.2. Injury Characteristics

Injury morphologies included Chance fractures and horizontal fractures such as posterior column fractures which encompassed pars, facet, and/or pedicle fractures with or without vertebral body fracture. Of the 11 patients reviewed, 5 sustained bony Chance fractures and 6 sustained posterior column fractures. 8 patients in this study presented with multilevel spine injury with 4 patients comprising 1 level fracture, 2 level fracture, or >2 level fracture (Table 1). All patients in the study were neurologically intact. No patient had posterior ligamentous complex injury that contributed to the assessment of a TLICS = 4 category.

### 3.3. Operative Characteristics

Stratified procedure level was recorded for each patient. In the MISF group, 3 patients received instrumentation from L1–L5. One patient underwent T8–T12 fixation. Two-level fusions were performed on 3 patients, followed by 4-level fusion on 1 patient. In the OPSF group, 1 patient received instrumentation from L1–L5. One patient underwent T5–T11 fixation and another from T2–T7. Two-level fusion was done in 1 patient and a 6-level fusion in 2 patients

**Figure 1:**
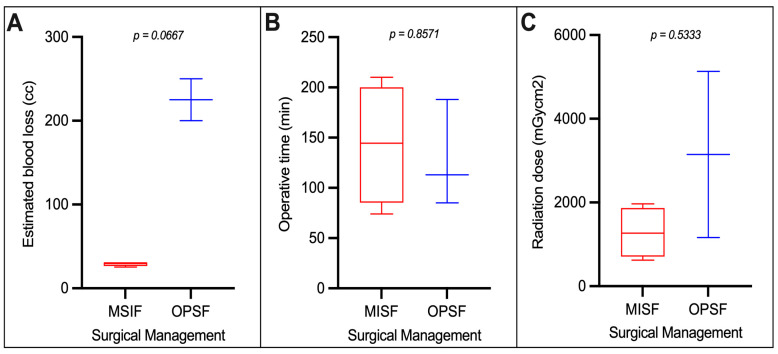
Comparison of surgical management techniques based on (**A**) estimated blood loss (cc), (**B**) operative time (minutes), and (**C**) total intraoperative radiation exposure (mGycm2). Mean scores and total range were plotted. *p*-values were calculated using Mann-Whitney test with significance set as *p* < 0.05.

Mean OR time and blood loss were collected for all operated patients. In the MISF group, the average OR time was 143 min with the average blood loss of 29 cc (Figure 1). In the OPSF group, the average OR time was 129 min and the average blood loss was 225 cc (Figure 1). Of all the patients who underwent surgical fixation, none required a blood transfusion following surgery to maintain a hemoglobin of >7 g/dL.

### 3.4. Radiation Exposure

Radiation exposure was collected following the use of fluoroscopic X-ray in the OPSF group, and both fluoroscopic X-ray and O-ARM CT in the MISF group. In the OPSF group, fluoroscopic X-ray radiation exposure average was 3145 mGycm2 (Figure 1). In comparison, radiation exposure in MISF group with total fluoroscopic X-ray radiation and O-ARM CT exposure averaged 1281 mGycm2 (Figure 1). In 1 patient O-ARM radiation exposures was not recorded due to missing records in the MISF group.

### 3.5. Complications

**Figure 2:**
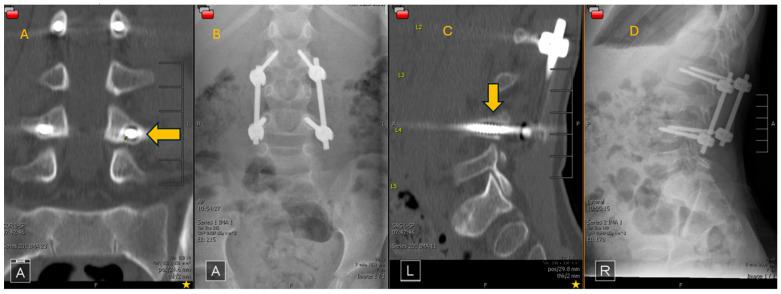
Post-operative CT and upright X-ray lumbar films. (**A**) AP film showing haloing around the left L4 screw, (**B**) AP representative X-ray, (**C**) Sagittal film focused on left L4 screw with arrow showing haloing, (**D**) Sagittal representative X-ray.

Complications were assessed by dividing patients into 3 age groups: <5 years (pre-school age), 5–13 (school age) and 14–17 years (adolescent). These age groups reflect the development process in children as their bone and muscle mature [16,19]. There were no complications in either group in the acute post-operative period. Hardware failure was also noted only in the MISF group outside of the acute peri-operative period, with 1 patient in the pre-school age category (<5 years) experiencing hardware failure (Figure 2).

There were no other complications reported including post-operative wound complications or infections, persistent pain, unexpected return to the OR, readmission, deep-vein thrombosis, or neurological deficits.

### 3.6. Length of Stay

LOS was recorded for all 11 patients, with an average of 9.5 days. For bracing, MISF, and OPSF group the average length of stays was 9.8, 9, 9.7 days respectively.

### 3.7. Alignment

All patients underwent 6–8 weeks of bracing whether in the bracing group or in the MISF/OPSF group post-operatively. Pre- and post-operative alignment of the thoracic and/or lumbosacral spine was recorded for 11 patients with mean follow-up of 4.8 months. Stable alignment was defined as having no exaggerated focal kyphosis around the fracture level. A change in the Cobb angle is only considered significant if it is greater than 5 degrees when compared to pre-operative measurements.

**Figure 3:**
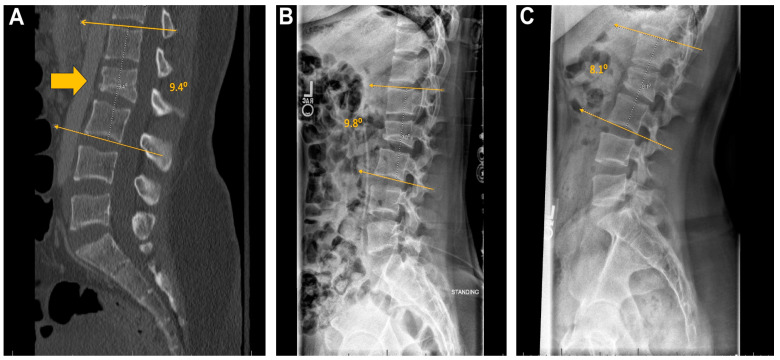
Example spinal alignment assessment in a patient managed with a brace. (**A**) CT-Lumbar spine with para-sagittal view demonstrated fracture morphology (arrow) at L2. Standing X-rays with lateral views at post-injury with brace (**B**) day 5, and (**C**) ~3-months.

**Figure 4:**
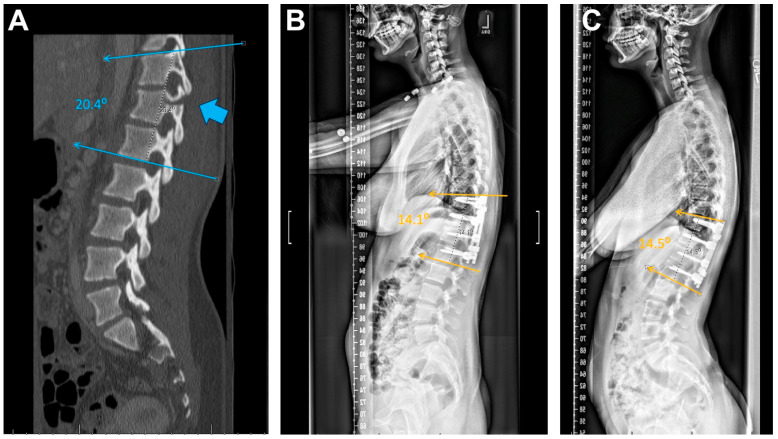
Example spinal alignment assessment in a patient managed with open screw fixation. (**A**) CT-thoracolumbar spine with parasagittal view demonstrating fracture morphology (arrow) at T12. Standing X-rays with lateral views at post-operative day 1 (**B**), and >2-year (**C**).

**Figure 5:**
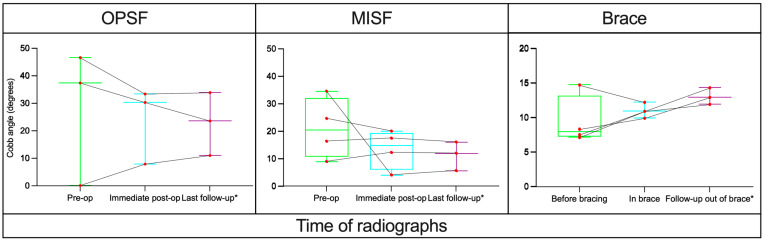
Case by case comparison of cobb angles over time across all management techniques. * Average last follow-ups were 4, 6, and 4.8 months respectively.

For the bracing group, 3 patients had stable alignment with 1 patient demonstrating worsened alignment (Figure 3). In the MISF group, 3 patients showed stable alignment, 1 had improved alignment, and none had worse alignment when upright in a brace were compared to presenting CT scans. Lastly, in the OPSF group, 3 had improved alignment and none had worse alignment when upright X-rays were compared to presenting CT scans (Figure 4A–C and Figure 5).

## 4. Discussion

This study reviewed a case series of pediatric patients who underwent non-operative or operative management, via MISF or OPSF, for traumatic TL fractures that fell into the category of having a TLICS of 4. The relative efficacy of each treatment modality was assessed with variables of interest being average OR time, blood loss, LOS, post-operative alignment, complication rate, and radiation exposure.

### 4.1. Injury Characteristics

When comparing the 3 treatment modalities, a fracture pathology pattern was appreciated. Of the 4 patients treated with braces, 3 had only posterior column fracture morphologies, and 1 had a Chance fracture. Overall, braced patients had the highest number of posterior column fractures. On further investigation of each of these cases, they were all horizontal fractures of the posterior elements and fell into the distraction fracture morphology making it receive the TLICS = 4 value. Most fractures were unilateral, traversing the pedicle or facet at times, likely adding to the fact it was managed conservatively in a brace. Using the Denis classification system, this would technically account to a single column fracture which could speak to its possible stability along with the fact that the ligaments were intact. This approach to management does not stray from the present literature which granted largely speaks to cervical unilateral non-displaced facet fractures, but it points out that most successfully can be managed in a collar [20]. Making a reasonable inference from this it is thus rational to interpret a similar approach can be taken when these fractures are occurring in the TL spine. Some literature surrounding thoracolumbar unilateral pedicle fractures does corroborate the efficacy of bracing for these injuries [1,3,5,21]. Future studies should evaluate this finding and see if similar outcomes persist in larger cohorts over multiple centers.

Conversely, both MISF and OPSF surgery groups possessed higher number of Chance type fractures. These findings are consistent with the spine trauma management guidelines that recommend surgical intervention for the management of Chance fractures which are considered unstable TL fractures needing fixation to maintain spinal stability and preventing neurological complications [4,5]. Literature does exist arguing for the management of these conservatively with a brace when approximated, dating back to the 1990′s [12], but recent literature, analyzing results over several level 1 trauma centers found better clinical outcomes when Chance fractures were managed surgically [22]. This was consistent with our findings.

### 4.2. Clinical Exam

In our patient cohort, neurological status played a pivotal role in determining the appropriate interventions. Non-operative management (brace) was selected based on injury morphology and the patient’s neurological status. This reflects an individualized, patient-centered approach that depends on the mechanism of injury and the patient’s neurological status. It prioritizes a non-invasive treatment (minimal potential risks/complications) over surgery when appropriate and addresses each patient’s clinical need based on their overall clinical status.

### 4.3. Radiation Exposure

When taking into consideration the surgical approach used, levels of radiation exposure were found to be the least with bracing and the highest with OPSF. These findings support the notion that both MISF and OPSF will carry some risk for radiation exposure, an especially important consideration in pediatric spine surgery [2,11]. Increasing radiation exposure can be a relevant risk factor for the development of childhood cancer, but our study shows that radiation levels attributable to O-ARM use during MISF cases is consistent with published standards on effective doses in our patient population [9]. Additionally, within our cohort, MISF appears to confer an advantage over OPSF, although not significant, with regard to radiation exposure likely attributable to the high of plain fluoroscopy in OPSF.

### 4.4. OR Time and Blood Loss

Length of operation and intraoperative blood loss are important surgical considerations in the pediatric population as well. Length of operation is directly correlated to time under anesthesia, and there is a growing body of literature supporting the association of deleterious effects of early exposure to anesthesia with neurodevelopmental impairment in children [8,10]. Moreover, the U.S. Food and Drug Administration has issued a warning regarding the prolonged use of general anesthesia in children younger than 3 years old [13]. Our patient population underwent surgery for an average of 143 min for MISF cases and 129 min in OPSF, both representing an increased risk of anesthesia-related injury when compared to bracing. The mean age of each group receiving surgery would not qualify for these warnings, but operative time should remain an important consideration when assessing holistic post-surgical improvement.

Finally, operative blood loss is another important secondary consideration when assessing a patient’s surgical candidacy. Blood loss has been associated with increased complication rates of infection, hematologic cross-reactions, and thrombotic events, which can impact recovery, hospital length of stay and mortality [15]. Our MISF and OPSF patients experienced an average blood loss of 29 mL and 225 mL respectively. This reflects the known benefits of MISF, with regard to blood loss, and further supports the safety of this modality in managing these injuries in this patient population [14].

### 4.5. Length of Stay and Complications

LOS was equal between all 3 groups. Follow-up was more longitudinal for patients managed surgically, likely a result of clinical protocols established to monitor implants. Clinic follow-up also detected 1 hardware failure at 8 months identified in a patient treated with MISF. There were no complications recorded for either bracing or OPSF. The younger population age may be a contributing factor for these failures, as younger patients have lower bone density, most likely attributable to the effects of puberty [23].

### 4.6. Alignment

Lastly, one important metric by which these treatment modalities can be measured is maintenance or correction of alignment. A change was only considered significant if the Cobb angle change was greater than 5 degrees compared to pre-operative measurements. For the bracing group, 75% of patients showed stable alignment over follow-up period of up to 8 months. By comparison, MISF and OPSF had 25% and 100% of patients showed improved alignment post-operatively. Notably, no patients in either surgical group demonstrated worsening of spinal alignment through their follow-up time period. This is likely explained by the mechanics of each surgery itself, which uses the rod-screw construct to restore pre-trauma spinal architecture, whereas patients who received braces did not have the same corrective adjustment, either in their hospital stay or after discharge. These findings are consistent with studies showing that surgical management of TL fractures can be superior to bracing in the correction of alignment in adults [12,17,24].

### 4.7. Limitations

The limitations of this study are inherent to the design of a case series. Small sample size, lack of ideal comparisons between groups, and in our study, procedural differences between surgeons limit the ability to draw strong conclusions. Future studies aiming to assess a difference between treatment modalities should ideally incorporate a prospective approach with a larger sample size, nursing that groups are matched accordingly. One suggestion for such a trial could be to assess differences between MISF and OPSF in the management of unstable fracture, where surgery is seen as superior to conservative management. In addition, validated metrics for pain and functional assessments would also provide a more comprehensive understanding of post-treatment outcomes. This was beyond the scope of our current analysis due to the inherent limitation of retrospective studies, which depends on the availability of recorded data. Hence, prospective studies with specific data collection strategies are recommended for future research endeavors. The study of a single institution primarily reflects the practice patterns and outcomes particular to this specific setting, which may limit the generalizability of our results to other centers. Future studies should aim to replicate and extend this work in multicenter studies, which would provide a more robust analysis of the efficacy and safety of the treatments discussed. Additional limitations lay inherent to the patient population in that there are no controls for demographic variables, fracture location or morphology, or follow-up duration. It is possible that these play a significant role in both the physiologic and socioeconomic post-surgical outcomes as well.

## 5. Conclusions

This case series provides an introductory perspective on the safety and efficacy of bracing, MISF, and OPSF in managing pediatric thoracolumbar fractures in the trauma setting. Surgery was associated with increased exposure to radiation, increased exposure to general anesthesia, and intraoperative blood loss. Surgical was management resulted in improved alignment for many patients, whereas bracing did not. These results suggest that surgery, both minimally invasive and open, are viable management options for pediatric TL fractures, taking into consideration their associated benefits as well as risks.

## Figures and Tables

**Table 1:** Demographics and clinical information for patients by treatment group.

	Operative		Non-Operative	Total	*p*-Value
	OPSF	MISF			
**Number of patients**	3	4	4	11	
**Mean age (years)**	16	10.5	10.25	11.9	0.052
**Gender**					0.77
Female	1	1	2	4	
Male	2	3	2	7	
**Race**					>0.99
White	3	2	2	7	
Asian	0	1	1	2	
Other	0	1	1	2	
**Ethnicity**					0.055
Hispanic	0	0	3	3	
Non-Hispanic	3	4	1	8	
**Mean height (cm)**	167.6	151.6	142.85	151.3	0.78
**Mean weight (kg)**	63.7	37.5	49.7	49.1	0.17
**Injury type**					>0.99
MVC	2	2	2	6	
ATV	0	1	0	1	
Fall	1	1	0	2	
Auto vs. Peds	0	0	1	1	
Penetrating injury	0	0	1	1	
**Fracture level location**					0.39
T2–T8	2	0	2	4	
T9–T12	0	1	0	1	
L1–L5	1	3	2	6	
**Morphology**					0.76
Chance fracture	2	2	1	5	
Posterior column fracture	1	2	3	6	
**Number of fractured levels**					0.34
1	1	2	1	4	
2	0	1	3	4	
>2	2	2	0	4	
**Polytrauma**	3	4	3	10	0.6
Mobility-limiting	0	0	1	1	
Not mobility-limiting	3	4	2	9	

*p*-values were calculated using Kruskal Wallis test for age, height, and weight. Gender, race, ethnicity, injury type, fracture level location, morphology, number of fractured levels and polytrauma were analyzed using Fisher’s exact test. Significance was set as *p* < 0.05. MVC—motor vehicle collision, ATV—all terrain vehicle, auto vs. peds—automobile versus pedestrian, mobility limiting trauma includes extremity and pelvic fractures.

## Data Availability

Data is contained within the article.
